# A pioneering transition program for adults with phenylketonuria – initial findings from a single-center one-year experience from Poland

**DOI:** 10.1016/j.clinsp.2026.101162

**Published:** 2026-09-23

**Authors:** Michal Kania, Martyna Pietruś, Łukasz Pawliński, Karolina Orchel-Szastak, Przemko Kwinta, Maciej T. Małecki

**Affiliations:** a Department of Metabolic Diseases and Diabetology, Jagiellonian University Medical College, Krakow, Poland; b University Hospital, Krakow, Poland; c European Reference Network for Hereditary Metabolic Diseases, MetabERN, Udine, Italy; d Department of Medical Genetics, Department of Pediatrics, Jagiellonian University Medical College, Krakow, Poland; e Department of Pediatrics, University Children’s Hospital, Krakow, Poland; f Department of Pediatrics, Jagiellonian University Medical College, Krakow, Poland

**Keywords:** Hyperphenylalaninemia, Phenylketonuria, PKU, Transition

## Abstract

Background In August 2024, at the University Hospital in Krakow, we initiated, for the first time in Poland, the transition of individuals with hyperphenylalaninemia to the adult metabolic center. We evaluated the experience and outcomes during the first year of this transition program.

Methods We included 120 adults with hyperphenylalaninemia who were followed between August 1st, 2024, and July 31st, 2025, in the Outpatient Metabolic Clinic at the University Hospital in Krakow, Poland.

Results The mean age of individuals was 31.77±10.80 years (range 18‒68); 69 (57.5%) were females. The majority, 101 (84.2%), were individuals with classic Phenylketonuria (PKU). Twenty (17.7%) individuals were off-diet, and a further 38 (33.6%) reported poor adherence. Participants were followed for a median of 5.6 (3.5‒7.7) months. The mean DBS Phe concentration from the 5-year period preceding the transition was 717.00±350.40 µmoL/L, with a mean percentage of results > 600 µmoL/L of 57.0 ± 41.4%. The mean Phe concentration during the follow-up period was 689.40±373.20 µmoL/L, with 55.5 ± 44.2% of Phe measurements exceeding the target value of 600 µmoL/L, revealing no significant change (p = 0.868 and 0.754, respectively). Seven (26.9%) children of transitioned individuals showed symptoms of maternal PKU.

Conclusions The initially poor Phe control in the cohort studied remained unchanged after transferring to the adult center, emphasizing that maintaining a restrictive diet in adults with PKU is challenging. Alarmingly high prevalence of unplanned pregnancies and maternal PKU highlights critical reproductive health concerns in this population. A longer follow-up is needed to assess the impact of the transition on the metabolic control of Phe.

## Highlights

• One year of pioneering adult PKU transition efforts in Poland was described.
• Phe control remained inadequate and unchanged after transition.
• Long-term adherence to the restrictive PKU diet remains challenging.

## Introduction

The necessity for consistent, lifelong care for individuals with Inherited Metabolic Diseases (IMDs), such as Phenylketonuria (PKU; OMIM 261,600), is increasingly acknowledged and demands further attention and development^1^^,^^2^ Current guidelines emphasize the importance of permanent treatment and medical follow-up to achieve the best possible neurological outcomes and prevent comorbidities^1^ Additionally, as more individuals with PKU reach reproductive age, preventing maternal PKU syndrome becomes increasingly crucial^1^ The estimated prevalence of PKU in Poland is 1 per 7000 live births, translating into about 50–60 live births with PKU per year^3^ According to the data from the time period 2009 to 2016, a total of 2706 patients with PKU were registered in the national healthcare provider database^3^ With such a large and ever-growing population of patients, just with PKU, the long-term care of patients with inborn metabolic errors would become impossible solely within pediatric centers.

Thus, the transition of adolescents from pediatric to adult health care is of utmost importance, but at the same time, it is a very challenging process^4^^,^^5^ Individuals suffering from chronic diseases encounter new responsibilities for their own health care, requiring more personal initiative and independence in managing their disease. In this situation, they are at risk of dropping out of medical care^6^ There is a large body of evidence in support of this, for i.e., type 1 diabetes, with descriptions of multiple challenges to this process and strategies to overcome them^7^

Until recently, the majority of adults with PKU have been treated in pediatric centers; however, throughout the previous 10‒20 years, some specialized adult centers have been established worldwide^4^^,^^5^^,^^8^, ^9^, ^10^ The evidence for an effective transition in IMDs, including PKU, is slowly emerging, with limited reports from the UK, Germany, Austria, and Portugal,^4^^,^^5^^,^^8^^,^^9^ which describe both obstacles and successes in this process. To date, there have been no reports on the care of adults with PKU in Poland, as the treatment was continued in pediatric centers. At the Outpatient Metabolic Clinic of the Department of Diabetology, Internal and Metabolic Diseases, University Hospital in Krakow, we initiated, for the first time in Poland, the transition of patients with PKU to an adult metabolic center.

This study aimed to describe the initial experience of the transition of patients with different forms of hyperphenylalaninemia to adult specialized metabolic care in Poland and to assess any potential changes in the metabolic control during the 1-year time period following the transition.

## Methods

### Population

For this clinical observation, we included 120 adults with hyperphenylalaninemia who were admitted to the Outpatient Metabolic Clinic of the Department of Diabetology, Internal and Metabolic Diseases, at the University Hospital in Krakow, Poland ‒ a tertiary referral center for patients with IMDs in southeastern Poland. The patients were admitted between August 1st, 2024, and July 31st, 2025. Patients were followed during this time period.

The inclusion criterion was a diagnosis of hyperphenylalaninemia in medical history (biochemical or genetic). Genetic testing results were available for the majority of patients (110/120)^11^ with a subset of patients in whom diagnosis was made only by biochemical testing (10/120). Individuals with untreated Phe concentrations during screening below 360 µmoL/L and not receiving treatment within one month after birth were regarded as having mild hyperphenylalaninemia (regardless of a potential future treatment initiation). Individuals with untreated Phe concentrations 360‒1200 µmoL/L were considered to have mild PKU, and with untreated Phe concentrations > 1200 µmoL/L as classic PKU.

Demographic and socioeconomic data, along with medical history, were recorded, including, but not limited to, age, sex, education level, occupation and employment, history of comorbidities and pharmacotherapy at baseline, genetic testing results, family history, including information on parenthood and offspring, and Maternal PKU [MPKU] syndrome in children. Data on the diagnosis of PKU and lifetime treatment were collected, including diagnoses made before or through the newborn screening program, delayed diagnosis and/or treatment, and treatment discontinuation (i.e., according to previous recommendations). New diagnoses of hypertension, diabetes, and hyperlipidemia during the follow-up period were recorded.

Hypertension was defined as 1) A history of treatment of hypertension or 2) At least two measurements of blood pressure ≥ 140/90 mmHg during medical appointments in a period of at least 4-weeks or ≥ 135/85 mmHg in home blood pressure monitoring. Hyperlipidemia was defined as 1) A history of treatment of hyperlipidemia or 2) Low-Density Lipoprotein (LDL) cholesterol ≥ 3 mmoL/L.

Lifetime Dried Blood Spot (DBS) Phe concentrations were recorded from the central Polish electronic database (PKUWeb) and multiple measures of metabolic control were calculated ‒ DBS Phe concentration on the day of transition, the number of Phe measurements per 1-month from the 1-year period preceding transition, mean DBS Phe concentrations from the 1-year and 5-year periods preceding transition, percentages of DBS Phe results > 600 µmoL/L during the 1-year and 5-year periods preceding transition, and the number of patients with all Phe measurements < 600 µmoL/L during the 1-year and 5-year periods preceding transition. For the follow-up period of this study, the number of Phe measurements per 1-month, the mean DBS Phe concentration, the percentage of DBS Phe measurements > 600 µmoL/L, and the number of patients with all Phe measurements < 600 µmoL/L were recorded.

The therapeutic ranges of Phe concentration, 120‒600 µmoL/L (2‒10 mg/dL) for adults with PKU, were based on the European Society for the Study of PKU (ESPKU) guidelines^1^^,^^2^

Baseline nutritional data, including Phe tolerance, daily protein requirements (total and per body weight), and daily amount of protein from amino acid formula were recorded. During the follow-up, the dietary recommendations were reviewed and reported. During the nutritional interview, data were gathered regarding self-reported adherence to dietary and amino acid formula consumption recommendations.

A comprehensive physical examination was conducted, and selected baseline parameters, including weight, height, and Body Mass Index (BMI), were documented.

Plasma amino acid profile was assessed, and phenylalanine and tyrosine concentrations on the day of transition were extracted.

### Statistical analysis

Adults with PKU were divided into two groups – early-treated PKU (ETPKU) and Late-Treated PKU (LTPKU). ETPKU was defined as treatment initiation within four weeks following birth,^1^ with the remaining individuals regarded as LTPKU. ETPKU and LTPKU were compared. Additionally, metabolic control was compared between the 1-year period preceding the transition and the follow-up period (0‒12 months, depending on the date of transition – August 1, 2024, to July 31, 2025). Finally, individuals with different levels of adherence to dietary recommendations were compared (four categories: complete adherence, good/almost complete adherence [small snack of unrecommended products several times weekly], poor/partial adherence [snacks of unrecommended products multiple times weekly and/or consuming meat, dairy, wheat products, rice, etc., multiple times weekly], no PKU diet at all).

A beta regression model was built to identify factors significantly impacting the mean percentage of DBS Phe measurements > 600 µmoL/L during the last 5-year period preceding the transition. The selection of variables for the model was based on the exploratory analysis of data and previous reports.

All analyses were performed using SPSS ver. 10 and Jamovi version 2.7.2. For normally distributed data, means and Standard Deviations (SDs) were calculated. For non-normally distributed data, medians and Interquartile Ranges (IQRs) were calculated. Normality was assessed with the Shapiro-Wilk test. Normally distributed data were analyzed using the Student *t*-test or the pairwise *t*-test, and non-normally distributed data were analyzed using the Mann-Whitney *U test* or the Wilcoxon rank sum test. Categorical unpaired data were analyzed using the chi-squared test or McNemar’s test. The Bonferroni method was used to correct for multiple comparisons when > 2 groups were compared. Due to the exploratory nature of this study, no sample-size calculation was performed. A p-value < 0.05 was regarded as statistically significant.

### Ethics

This study was approved by the Institutional Review Board – the Jagiellonian University Bioethics Committee, approval number 1072.6120.111.2024. The study was conducted in accordance with local legislation and institutional requirements. As this was a retrospective analysis of patients' data with two changes to the standard of care, obtaining written informed consent to participate in this study was not mandatory. The study design and reporting were done according to the Strengthening the Reporting of Observational Studies in Epidemiology (STROBE) guidelines.

## Results

Between August 1, 2024, and July 31, 2025, 120 adult individuals with various forms of hyperphenylalaninemia were transitioned to our center. The majority 115 (95.8%) of individuals were transitioned from the Children's University Hospital in Krakow, Poland. Of the remaining individuals, 3 (2.5%) moved from another metabolic care center. In 2 (1.7%) individuals, the treatment of PKU was interrupted by the age of 18 according to the earlier, historical recommendations, and now they returned under the PKU medical care.

The mean age of the individuals was 31.77±10.80 years, ranging from 18 to 68 years. Of this number, 55 (45.8%) individuals were aged 18‒29 years, 58 (48.3%) 30‒49 years, 6 (5.0%) 50‒65 years, and 1 (0.8%) > 65-years. Of 120 adults with hyperphenylalaninemia, 69 (57.5%) were females. LTPKU were older than ETPKU individuals (42.5 [37.8‒45.3] vs. 27.0 [21.0‒33.0] years, p < 0.001).

Based on medical records, there were 101 (84.2%) individuals with classical PKU, 11 (9.2%) with mild PKU, and 8 (6.7%) with hyperphenylalaninemia. A large majority of individuals 115 (95.8%) were currently treated or had a history of treatment initiation before treatment interruption. None of the participants was treated with BH4 or pegwaliase. Of 5 untreated cases, 4 had hyperphenylalaninemia with lifetime Phe values < 360 mmoL/L, and 1 had values between 360 and 600 mmoL/L. In 96 (80.0%) individuals, hyperphenylalaninemia/PKU was diagnosed during newborn screening. There were 90 (75.0%) individuals classified as ETPKU and 30 (25.0%) as LTPKU.

In total, 217 pathogenic variants of the *PAH* gene were identified in 109 (90.8%) individuals (Fig. 1). The most common pathogenic variant identified was R408W 137 (63.1%), followed by R158Q 8 (3.7%), P281L 6 (2.8%), IVS10nt-11_g>a 6 (2.8%), and L348V 5 (2,3%), with the prevalence of the remaining variants lower than 5. The most common genotype was R408W homozygote in 43 (35.8%) individuals.

**Fig. 1:**
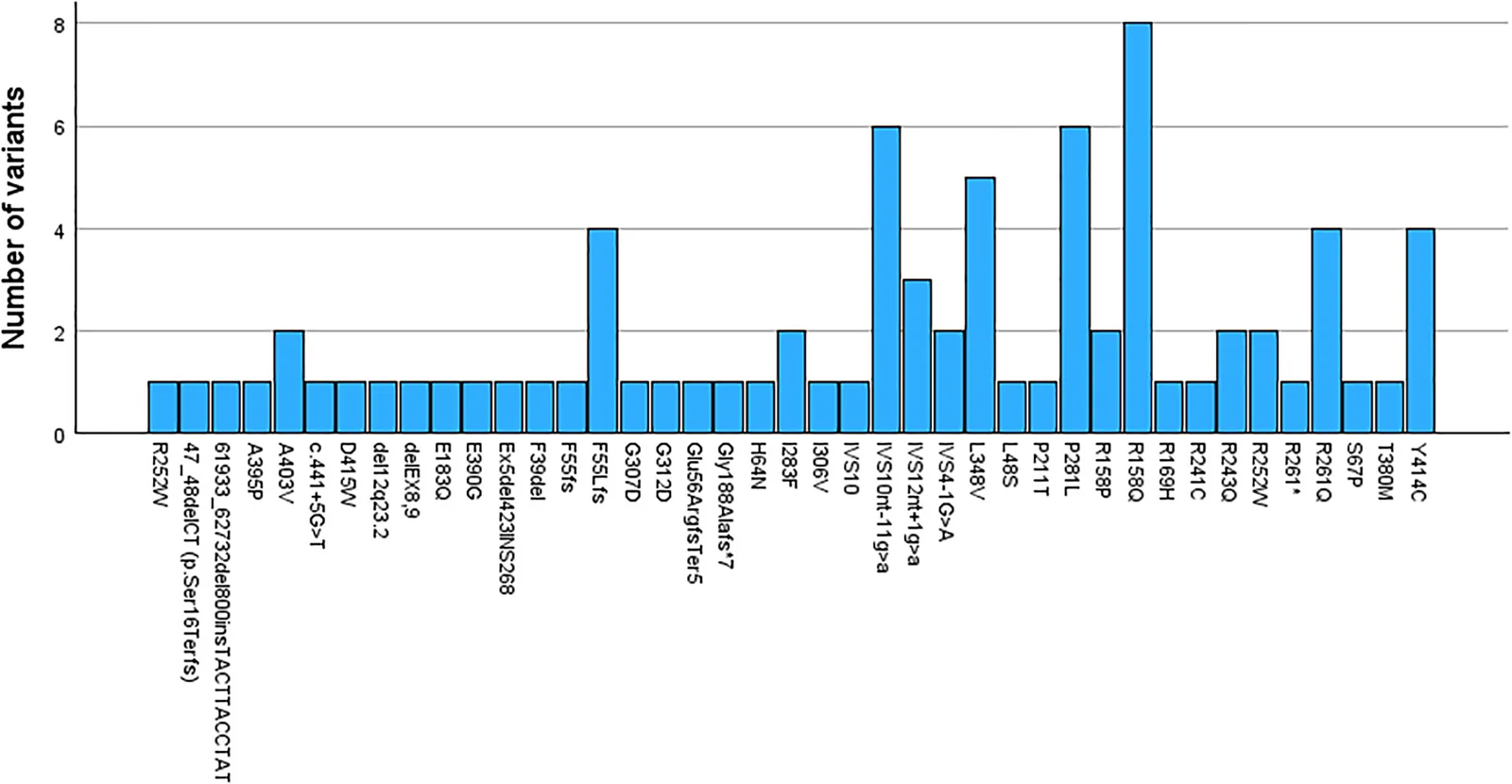
Pathogenic variants of the PAH gene in the study population (R408W excluded). The most common pathogenic variant identified was R408W 137 (63.1%), not shown on the graph for visual clarity.

### Comorbidity burden

The mean BMI of participants was 26.07±6.01 kg/m^2^, with 35 (30.7%) classified as overweight (BMI 25‒29.9 kg/m^2^) and 23 (20.2%) individuals classified as obese (BMI ≥ 30 kg/m^2^). The most common comorbidities (history before transition or diagnosed during the follow-up period) included (Table 1): hyperlipidemia 17 (14.2%), hypertension 11 (9.2%), hypothyroidism 8 (6.7%), epilepsy 12 (10.0%), anxiety disorders 15 (12.5%), and depressive disorder 7 (5.8%). Twelve cases of hyperlipidemia, 1 case of hypertension, and 1 case of hypothyroidism were diagnosed during the follow-up period. Mental disability, epilepsy, arterial hypertension, and hyperlipidemia were more common in LTPKU than in ETPKU (Table 1). MPKU syndrome was previously diagnosed in 2 participants.

**Table 1:** Population characteristics.

	All participants	ETPKU	LTPKU	p for ETPKU vs. LTPKU
**Age [yr]**	31.77±10.80	27.0 (21.0‒33.0)	42.5 (37.8‒45.3)	<0.001
**Gender (female)**	69 (57.5%)	54 (60.0%)	15 (50.0%)	0.337
**BMI [kg/m^2^]**	26.07±6.01	24.65 (21.78‒28.07)	26.22 (24.30‒28.73)	0.096
**BMI category**				0.546
< 18.5 kg/m^2^	6 (5.3%)	5 (5.7%)	1 (3.7%)	
18.5‒24.9 kg/m^2^	50 (43.8%)	41 (47.1%)	9 (33.3%)	
25‒29.9 kg/m^2^	35 (30.7%)	24 (27.6%)	11 (40.7%)	
30 kg/m^2^	23 (20.2%)	17 (19.6%)	6 (22.2%)	
**Comorbidities**				
Mental disability	33 (27.5%)	8 (8.9%)	25 (83.3%)	<0.001
Anxiety disorders	15 (12.5%)	9 (10.0%)	6 (20.0%)	0.200
Depressive disorder	7 (5.8%)	6 (6.7%)	1 (3.3%)	0.679
Epilepsy	12 (10.0%)	3 (3.3%)	9 (30.0%)	<0.001
Hyperlipidemia	17 (14.2%)	8 (8.9%)	9 (30.0%)	0.007
Diabetes	1 (0.8%)	0	1 (3.3%)	0.250
Arterial hypertension	11 (9.2%)	4 (4.4%)	7 (23.3%)	0.005
Hypothyroidism	8 (6.7%)	5 (5.6%)	3 (10.0%)	0.678

PKU, Phenylketonuria; ETPKU, Early-Treated PKU; LTPKU, Late-Treated PKU; BMI Body Mass Index.

### Socio-demographic data

The majority of participants originated from and lived in south-eastern Poland. The mean distance from the place of living to the Center was 115.37±78.33 kilometers and ranged from 3 to 348 kilometers. The educational level of individuals with PKU is presented in Table 2. LTPKU more frequently and almost exclusively had no education, only primary education, or attended special schools, whereas university education was virtually exclusive to ETPKU (Table 2). Current employment was reported by 57 (50.0%) individuals, 8 (7.0%) had worked at some point in their life, and 49 (43.0%) had never been employed. The history of current employment in LTPKU was less common than in ETPKU (Table 2).

**Table 2:** Socio-demographic data of the study population.

	All participants	ETPKU	LTPKU	p for ETPKU vs. LTPKU
**Educational level**				<0.001
No education	12 (10.1%)	2 (2.2%)	10 (33.3%)	
Special school	5 (2.5%)	4 (4.5%)	7 (23.3%)	
Primary education	3 (2.5%)	0	3 (10.0%)	
High school/Vocational school	44 (38.0%)	36 (40.5%)	8 (26.6%)	
High school/Vocational school ‒ Ongoing	13 (10.9%)	13 (14.6%)	0	
University degree	22 (18.5%)	20 (22.5%)	2 (6.7%)	
University ‒ Ongoing	14 (11.8%)	14 (15.7%)	0	
**Employment**				0.017
Never	49 (43.0%)	30 (35.3%)	19 (65.5%)	
Current	57 (50.0%)	48 (56.5%)	9 (31.0%)	
Previous	8 (7.0%)	7 (8.2%)	1 (3.4%)	
**Parenthood**	18 (15.1%)	16 (18.0%)	2 (6.7%)	0.236

PKU, Phenylketonuria; ETPKU, Early-Treated PKU; LTPKU, Late-Treated PKU.

### Offspring and pregnancy outcomes

Eighteen individuals had 26 children; of these, 7 showed symptoms of MPKU syndrome (26.9%). Three children inherited PKU (with 2 of them also affected by MPKU syndrome). During the follow-up period, one unplanned pregnancy occurred in a woman who had been pregnant before her first visit to our center and was Lost To Follow-Up (LTFU) at the pediatric center with her last visit several years earlier.

### Metabolic control and adherence to treatment and protein requirement

DBS Phe concentration on the day of transition was 775.80±411.60 µmoL/L. The plasma Phe and tyrosine concentrations on the day of transition were 903.41±483.78 µmoL/L and 43.67±28.90 µmoL/L, respectively (Phe/Tyr ratio 25.97±16.77). In 38 (32.8%) individuals, Tyr level was below the lower limit of norm, in 69 (59.5%) it was within the norm, and in 9 (7.8%) it was higher than the upper limit of norm. The mean DBS Phe concentrations from the 1-year and 5-year periods preceding the transition were 708.60±381.60 and 717.0 ± 350.40 μmol/L, respectively. The percentages of DBS Phe results > 600 µmoL/L during the 1-year and 5-year periods before the transition were 54.7 ± 45.5% and 57.0 ± 41.4%, respectively. Twenty-nine (30.2%) and 13 (11.3%) patients had all Phe measurements < 600 µmoL/L during the 1-year and 5-year periods before the transition, respectively. No significant differences were found between ETPKU and LTPKU (Table 3).

**Table 3:** Metabolic control and self-reported adherence to treatment recommendations.

	All participants	ETPKU	LTPKU	p for ETPKU vs. LTPKU
DBS Phe concentration on the day of transition	775.80±411.60	731.4 (447.0‒1056.9)	807.6 (507.0‒10.48.2)	0.398
Plasma Phe concentration on the day of transition	903.41±483.78	854.45 (539.68‒1179.85)	926.90 (648.13‒1271.85)	0.364
Plasma tyrosine concentration on the day of transition	43.67±28.90	36.70 (29.80‒45.23)	32.25 (28.65‒43.65)	0.357
Mean DBS Phe concentration from the 1-year period before the transition	708.60±381.60	619.20 (384.60‒1020.6)	676.2 (453.6‒964.8)	0.506
Mean DBS Phe concentration from the 5-year period before the transition	717.00±350.40	703.8 (399.0‒994.2)	714.0 (480.0‒895.8)	0.659
Percentage of DBS Phe results > 600 µmoL/L during the 1-year period before the transition	54.7 ± 45.5%	50.0% (0‒100%)	85.7% (33.3‒100%)	0.308
Percentage of DBS Phe results > 600 µmoL/L during the 5-year period before the transition	57.0 ± 41.4%	75.0% (10.0‒100%)	78.7% (18.7‒100%)	0.453
All Phe measurements < 600 µmoL/L during the 1-year period before the transition	29 (30.2%)	24 (32.9%)	5 (21.7%)	0.310
All Phe measurements < 600 µmoL/L during the 5-year period before the transition	13 (11.3%)	13 (14.6%)	0	0.038
Self-reported adherence to amino acid formula recommendations				<0.001
Drinking all doses every day	70 (61.9%)	46 (54.8%)	24 (85.7%)	
Omitting some doses multiple times weekly	33 (29.2%)	32 (38.1%)	1 (3.6%)	
No amino acid formula consumption	9 (7.9%)	6 (7.1%)	3 (10.7%)	
Adherence to dietary recommendations				0.245
Complete adherence	31 (27.4%)	19 (22.6%)	12 (41.4%)	
Almost complete adherence (small snack of unrecommended products several times weekly)	24 (21.2%)	18 (21.4%)	6 (20.7%)	
Poor adherence (snacks of unrecommended products multiple times weekly and/or consuming meat, dairy, wheat products, rice, etc., multiple times weekly)	38 (33.6%)	31 (36.9%)	7 (24.1%)	
No PKU diet at all	20 (17.7%)	16 (19.0%)	4 (13.8%)	

PKU, Phenylketonuria; ETPKU, Early-Treated PKU; LTPKU, Late-Treated PKU, DBS, Dried Blood Spot, Phe, Phenylalanine.

Self-reported adherence to treatment recommendations is summarized in Table 3. No amino acid formula consumption was reported by 9 (7.9%) individuals, with an additional 33 (29.2%) omitting some doses multiple times weekly. Twenty (17.7%) individuals were not using any dietary restrictions, and a further 38 (33.6%) reported poor adherence to diet (for example, eating snacks of unrecommended products multiple times weekly and/or consuming meat, dairy, wheat products, rice, etc., multiple times weekly). Individuals with LTPKU appeared more adherent to amino acid formula consumption recommendations, with the majority, 24 (85.7%), consuming all doses every day (Table 3).

At baseline, the mean daily Phe tolerance as recommended by the previous center of care was 443.91±111.10 mg, with a mean daily protein of 65.41±7.16 g (0.94±0.19 g/kg body weight), of which 55.88±7.15 g was from the amino acid formula. After review in the current center, the new recommended Phe tolerance was 452.33±140.18 mg, with a mean daily protein of 67.55±6.61 g (0.97±0.19 g/kg body weight), of which 56.41±9.31 g was from the amino acid formula. The updated Phe tolerance did not change significantly (p = 0.265).

There were significant differences between individuals with different levels of adherence to dietary recommendations (Fig. 2). Individuals who did not follow the PKU diet had higher Phe concentrations, performed fewer Phe measurements, and had higher BMI than individuals who adhered to the recommendation completely (Fig. 2).

**Fig. 2:**
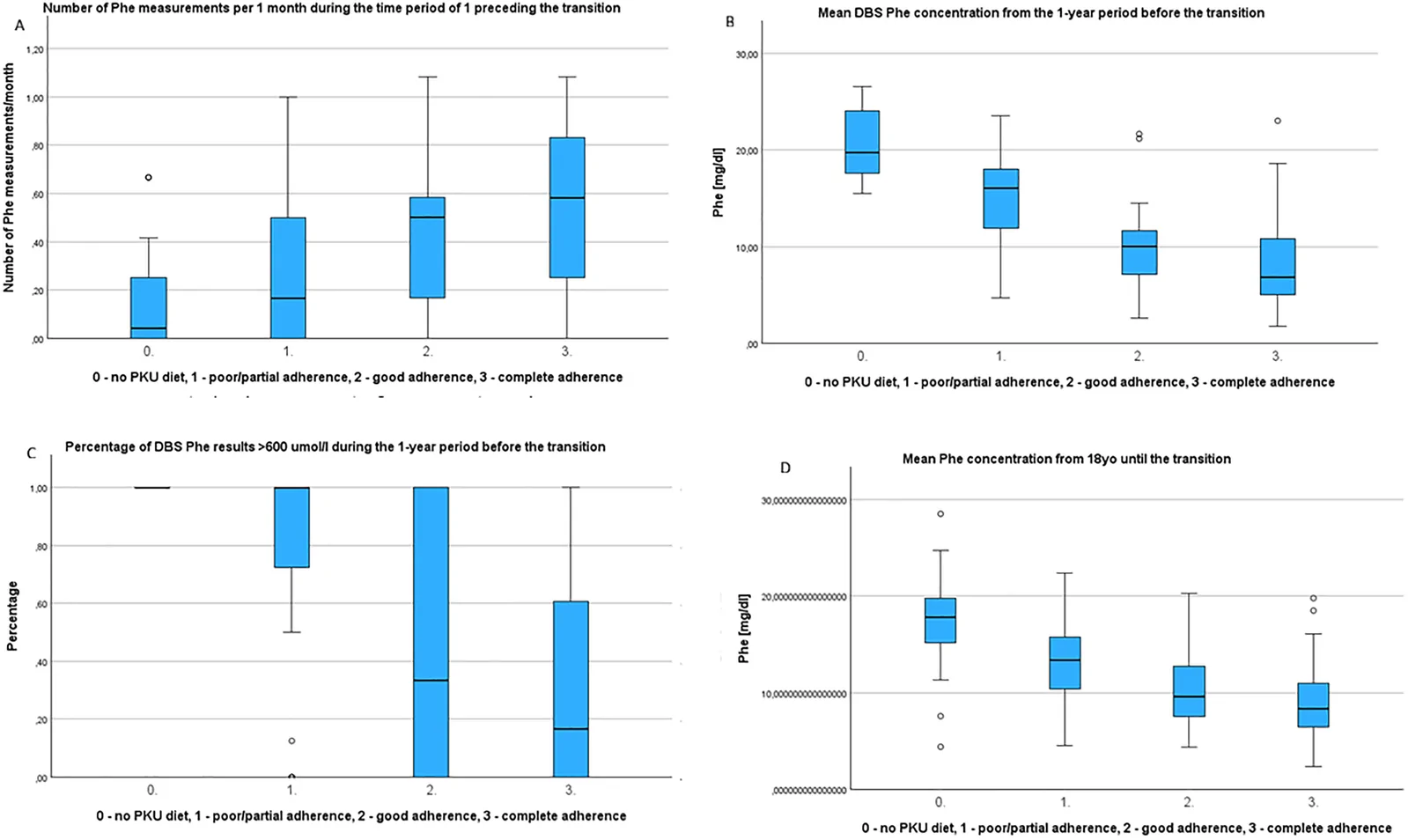
(A‒D) The impact of adherence to the PKU diet on metabolic control. Groups: 0 – no adherence to PKU diet, 1 – poor/partial adherence (snacks of unrecommended products multiple times weekly and/or consuming meat, dairy, wheat products, rice, etc., multiple times weekly), 2 – good adherence (small snack of unrecommended products several times weekly), 3 – complete adherence. (A) Tthe number of Phe measurements per 1-month during the time period of 1 preceding the transition was lower in group 0 vs. 2 (p = 0.009), 0 vs. 3 (p = 0.000), and 1 vs. 3 (p = 0.013). (B) Mean DBS Phe concentration from the 1-year period before the transition was higher in group 0 vs. 2 (p = 0.000), 0 vs. 3 (p = 0.000), and 1 vs. 3 (p = 0.000). (C) The percentage of DBS Phe results > 600 umoL/L during the 1-year period before the transition was higher in group 0 vs. 2 (p = 0.005), 0 vs. 3 (p = 0.000), and 1 vs. 3 (p = 0.001). (D) The mean Phe concentration from 18yo until the transition was higher in group 0 vs. 2 (p = 0.001), 0 vs. 3 (p = 0.000), and 1 vs. 3 (p = 0.003). All p values were adjusted for multiple comparisons.

In the beta regression model for factors significantly affecting the mean percentage of DBS Phe measurements > 600 µmoL/L during the last five-year period before the transition, which included age, sex, BMI, and ETPKU/LTPKU diagnosis, only the latter was not statistically significant (Table 4). Older participants, males, and individuals with a BMI ≥ 25 kg/m^2^ had a higher risk of having a greater percentage of Phe measurements exceeding 600 μmoL/L.

**Table 4:** A beta regression model for factors impacting the mean percentage of DBS Phe measurements > 600 umoL/L during the last 5-year period preceding the transition.

Factor	OR	95% CI	p
Age [years]	1.013	1.004 - 1.020	0.006
Sex (male vs. female)	1.280	1.105 - 1.480	0.001
ETPKU vs. LTPKU	1.196	0.979 - 1.500	0.080
BMI [≥ 25 kg/m^2^ vs. < 25 kg/m^2^)	1.310	1.131 - 1.510	<0.001

DBS, Dried Blood Spot; Phe, Phenylalanine; BMI, Body Mass Index; PKU, Phenylketonuria; ETPKU, Early-Treated PKU; LTPKU, Late-Treated PKU.

### Follow-up during the first year of care after the transition

The follow-up period began on the date of the first patient's transition (August 1, 2024) and lasted until July 31, 2025. During this period, 59 (49.2%) patients had at least one follow-up visit scheduled, as the transition occurred at various times throughout the year. The remaining patients had their first scheduled visits planned for the subsequent year. Of the planned 77 outpatient clinic follow-up visits, 70 visits were conducted in 54 (88.5%) patients. Attempts were made to contact five patients who missed follow-up visits; four of them rescheduled their visits after July 31, 2025. Only one patient did not respond and is considered LTFU during this period. Participants were followed for a median of 5.6 (3.5‒7.7) months (ranging from 0 to 12 months) after the transition. They performed 2.92±3.14 Phe measurements during this time period, with 94 patients performing at least one Phe measurement in this period. The mean number of Phe measurements per 1 month of the follow-up was 0.45±0.37. The mean Phe concentration during the follow-up period was 689.40±373.20 µmoL/L, with a mean of 55.5 ± 44.2% of Phe measurements exceeding the target value of 600 µmoL/L. Metabolic control of PKU during the follow-up period vs. the 1-year period before transition revealed no significant change (p = 0.868 and 0.754, respectively) and is summarized in Table 5.

**Table 5:** Metabolic control of PKU during the follow-up period vs. the 1-year period before transition.

	**1-year before transition**	**Follow-up period after transition**	**P for 1-year before transition vs. follow-up period after transition**
Number of Phe measurements per 1 month	0.38±0.33	0.45±0.37	0.110
Mean Phe concentration	708.6 ± 381.6	689.40±373.2	0.868
Percentage of DBS Phe results > 600 umoL/L	54.7 ± 45.5%	55.5%±44.2%	0.754
Number of patients with all Phe measurements < 600 umoL/L	29 (30.2%)	27 (28.7%)	0.804

DBS, Dried Blood Spot; Phe, Phenylalanine.

## Discussion

After reviewing the current published evidence, we consider this study to be the first to report on a large group of recently transitioned adults with PKU from Central Europe. It is also the first study to describe a population of adults with PKU in Poland so far.

According to the European guidelines for PKU, treatment and follow-up should continue through adulthood^1^^,^^2^ Until 2024, the care for adults with PKU in Poland had been provided solely by pediatric metabolic centers. In August 2024, our center was the first in Poland to initiate the transition of adult patients with PKU. According to a survey of centers within the European Reference Network for Hereditary Metabolic Disorders (MetabERN), transitional care in IMDs is still underdeveloped in Europe^12^ The care for adults with PKU in Europe varies regionally, with an overall low number of services dedicated to adults^10^ In selected countries, including the United Kingdom, France, Spain, Portugal, Italy, Germany, Slovenia, Czechia, Austria, and Hungary, specialist adult metabolic care centers have been set up, and varying transition protocols have been implemented^4^, ^5^, ^6^^,^^8^^,^^9^^,^^13^ A recent Italian study underlined the need for a specialized adult PKU care, with adults with PKU still treated in pediatric centers reporting psychological, emotional, and practical discomfort, leading to non-compliance or discontinuation of treatment due to the lack of such a setting^14^ A study from Austria identified 281 individuals born between 1966 and 1999 and diagnosed with PKU. Of this number, 177 (63.4%) were LTFU in 2020. LTFU was more common among males and older individuals^8^

As stated in a recent review of evidence,^1^^,^^6^ only two European studies reporting the experiences of specific management of transition of PKU patients from childhood to adulthood have been published^4^^,^^5^ An adult outpatient clinic for IMDs was established in Leipzig, Germany, and all patients with PKU aged 18-years and older were transferred from pediatric to adult care. The authors described the transition process^13^ and then a 10-year retrospective evaluation of 96 adults with PKU was described, revealing a successful transition with the metabolic control regarded by the authors as “satisfactory”^4^ A dedicated center was also established in Porto, Portugal, with the first patient transitioned in 2015. In a recent study, a cohort of 56 individuals with PKU, aged over 18-years, was described. These individuals were transferred from pediatric to adult care and followed for two years (2015‒2016)^5^

Understandably, adults with PKU should be treated in an adult environment, with healthcare providers experienced in providing care for adults and able to manage their issues and comorbidities^1^^,^^2^^,^^15^ Some adult patients have reported being discouraged by the pediatric care, discontinuing treatment, and are often unmotivated to resume specialist care^16^ This can result in deterioration of metabolic control. In our center, the transition was based on the ESPKU guidelines,^1^^,^^2^ although taking local circumstances into consideration.

Transition should be planned carefully to ensure that the patients and their family gain trust and establish a connection with their adult team^1^^,^^2^ During the transition process, which should start as early as 12-years old according to the ESPKU guidelines, individuals with PKU should gradually build their confidence in dealing with PKU, and possess skills and knowledge to take responsibility for their own treatment^1^^,^^2^ The transition to an adult treatment center typically takes place when the patient is between 16 and 18 years old,^1^^,^^2^ but given the decreased autonomy of some individuals with PKU, the timing of transition may differ. Although some data support better outcomes when the initial clinic visits are held together by both the pediatric and adult metabolic teams,^17^ this scenario may not be viable locally. Nevertheless, no standardized transition program exists.

At our center, the transition was preceded by training at the pediatric center of the University Children’s Hospital of the Jagiellonian University Medical College, which is the same founding body as our center, the University Hospital. Due to the large number of transitioned individuals during the first year and the limited number of physicians and nutritionists, it was not feasible for all individuals to perform shared pediatric/adult visits. The transition was always accompanied by a written letter providing complete information on the history of PKU treatment, supplemented by a telephone conversation or in-person consultation for more complicated cases.

During the transition visit, patients were consulted with a nutritionist and, when needed, a psychologist. Patients underwent blood testing (including, but not limited to, DBS Phe, plasma amino acid profile, lipids, electrolytes, protein and albumin, vitamins), a chest X-Ray, and a DEXA scan. Previous dietary recommendations were reviewed and adjusted based on current body weight (IBW was calculated) and energy expenditure, with the level of physical activity taken into account. For individuals with classic PKU, the first follow-up visit was planned within the next 1- to 6-months, with subsequent visits scheduled at approximately 6-month intervals. For individuals with mild hyperphenylalaninemia, the first follow-up visit was scheduled after approximately 12-months. Individuals with classic PKU were instructed to perform Phe measurements at home at least once monthly (except women planning pregnancy – then at least once weekly). Individuals with mild hyperphenylalaninemia were instructed to perform a Phe measurement at home at least once every quarter.

Of the transferred individuals with PKU who had their follow-up visits scheduled until July 31st, 2025, most (54 of 59, 88.5%) maintained contact with our center within the one-year follow-up period. The frequency of DBS Phe monitoring and DBS Phe levels were comparable between the follow-up period and the 1 one period preceding the transition. While the difference in the frequency of DBS Phe monitoring was not statistically significant (p = 0.110) between the two periods, this may represent a clinically meaningful direction of change towards its higher frequency. Previous studies showed similar results, with a limited impact of the transition on the metabolic control of PKU^4^^,^^5^ In one study, patients performed slightly more Phe measurements after the transition, which could be potentially explained by an increase in motivation following the transition^5^

Comparison of dietary recommendations from the pediatric clinic with the reviewed ones in our center showed an increase in total daily protein dose corrected for IBW; however, the change was relatively small (0.04 *g*/1 kg IBW). During the transition, patients’ activity levels were assessed, and daily protein and protein from formula were adjusted, which may have contributed to this increase.

A high percentage of patients remaining in follow-up post-transition in our cohort may reflect their motivation, as they had to plan their first visit themselves. Still, the fact that patients do not miss visits may reflect a good perception of the quality of care provided^4^^,^^5^ This high short-term retention rate is consistent with and slightly exceeds that of previously reported transition programs^4^^,^^5^ However, the real challenge of patient retention typically emerges at 2‒5 years post-transition.

Adults with PKU in our cohort presented with unsatisfactory metabolic control throughout previous years, with more than 50% Phe concentration measurements exceeding the therapeutic goal of 600 µmoL/L. During the observation period, metabolic control in transitioned individuals did not improve and remained unchanged, with a high frequency of Phe measurements exceeding target values. This might be seen as a failure of the transition process, but the observation period might have been too short to show a meaningful impact on metabolic control. Our observation is consistent with previous reports on the transition process, which also failed to demonstrate any improvement in metabolic control in adults with PKU. The aforementioned German study reported median 10-year Phe concentrations similar to ours (median 673.0 μmoL/L, range 213.0–1381.1)^4^ In the cited study from Portugal, median Phe levels were lower, and the proportion of results below 480 μmoL/L was higher than in our subgroup; however, the authors included younger individuals and excluded non-adherent ones,^5^ casting doubt on the robustness of this report. Another report from Italy indicated that nearly half of adults had a high plasma value over the past six months, attributing this to a lack of understanding of the condition and limited awareness of the long-term consequences of poor metabolic control in adulthood^18^ A regression model in our cohort identified older age, male sex, and overweight/obesity as factors independently associated with worse metabolic control of Phe. Research consistently demonstrates that metabolic control declines with age in patients with PKU. A recent multicenter European report revealed that the proportion of blood Phe measurements within the target range decreases from about two-thirds in individuals aged 19‒30 years to only 40% in those aged 41-years or older^19^ A study in the USA reported even more alarming data, revealing that only 49% of teenagers, 41% of young adults, and 31% of patients aged 30 and above maintained these levels^20^ A gender gap in care is evident, with higher rates of missed appointments and higher Phe levels in adult men, particularly young men, and a greater focus on women's reproductive health issues^8^

Although there is a lack of high-quality data from prospective studies, an increased frequency of blood Phe monitoring was reported to be associated with better metabolic control, including in adults^19^ The number of Phe measurements in our cohort was well below the endorsed once-monthly frequency, casting doubts on the applicability of this recommendation. The DBS Phe test is straightforward and can be performed at home by patients. As samples are sent to the laboratory afterward and, in Poland, the cost of packaging and delivery is covered by patients, this could be one of the driving factors behind the low frequency of DBS measurements. Nevertheless, reports from other countries where the cost of this process is state-covered reveal similar observations^19^ We hypothesize that the lack of patients’ awareness of its importance and the perceived lack of impact on their treatment could also explain this observation.

About 40% of our cohort reported either a regular diet without any protein restriction or only partial adherence to it. This number is higher than in previous reports from Germany (ca. 27% off diet)^4^ and Portugal,^5^ but lower than a cohort from the UK. In the latter, only 32% followed a Phe-restricted diet with the recommended intake of protein substitutes. Unsurprisingly, individuals who did not follow the PKU diet had significantly higher Phe levels and performed fewer Phe measurements. The high rate of non-adherence is particularly dangerous for women of childbearing age and poses a significant risk of MPKU syndrome in the event of an unplanned pregnancy. This underscores the urgency of developing effective interventions to improve dietary adherence in this vulnerable population. In line with previous reports^21^ the prevalence of overweight and obesity was also higher in the non-adherent group, potentially reflecting overall poor quality nutrition.

There are multiple reasons for such low adherence to dietary recommendations in adults with PKU. This is particularly true for individuals with classical PKU who have a low tolerance for Phe and natural protein, necessitating a strict diet even in adulthood, despite a moderate increase in Phe tolerance with age^22^ The genetic landscape of this PKU population has already been described^11^ The majority of patients had the classic PKU variant, possibly playing a role as a factor responsible for challenging adherence to recommendations. Phe-restricted diet with limited food choices, poor taste of protein substitutes, difficulties in food preparation, high cost of low-Phe products, and significant social restrictions contribute to this difficulty^10^^,^^14^^,^^23^

Our study population was too small and susceptible to selection bias, making it difficult to draw conclusions about a higher comorbidity burden than in the non-PKU population. A larger population or registry is required to address this evidence gap. Nevertheless, as a recent review reported, there is a higher risk of bone-related abnormalities, overweight/obesity, cardiovascular diseases, hypertension, COPD/asthma, diabetes, and gastrointestinal disorders,^24^ this matter requires attention. The prevalence of obesity was numerically lower than reported for the general population in Poland^25^ Hypertension, dyslipidemia, and diabetes were more frequent in the LTPKU group, as these patients were older. Newer reviews indicate that the previously reported risk of comorbidities in PKU may refer mostly to older LTPKU patients, with ETPKU patients being at higher risk of osteoporosis (not reported in our paper) and renal failure, with no cases in our cohort^26^ In our cohort, all patients with hypertension, and most with dyslipidemia, were > 45-years old (data not presented), and their etiology probably does not result from PKU.

Notably, a quarter of transitioned individuals had a mental disability, mostly due to LTPKU. This can be explained by the fact that the oldest individuals, who are the most affected, were transitioned during this initial phase of the process. Furthermore, although the pilot universal newborn screening program was launched in Warsaw in the late 1960s, followed by gradual implementation in other regions, it became universal only in 1994^27^ As a result, some cases were diagnosed late, even after 1990.

Unsurprisingly, LTPKU individuals were older, had a lower level of education, with university education or a degree being almost exclusive to the ETPKU group, and were less commonly employed than individuals with ETPKU. The higher prevalence of arterial hypertension and hyperlipidemia in LTPKU may result from more advanced age in this subgroup; however, some studies revealed that there is a higher burden of CVD in LTPKU, with no apparent pathomechanism^24^^,^^28^ In our cohort, LTPKU individuals were more likely to adhere to dietary recommendations, especially regarding the consumption of amino-acid formulas. Since most of the LTPKU individuals relied on their caregivers, this more accurately reflects the quality of care they received, including those in long-term care facilities, rather than their self-care, motivation, disease awareness, or the quality of transitional care. Still, according to the ESPKU guidelines, the transition and care should be individualized^1^^,^^2^ with LTPKU individuals with cognitive impairments and disabilities being the obvious candidates for such an approach.

PKU poses significant risks to both maternal health and neonatal development, making strict adherence to dietary management crucial for maintaining optimal maternal Phe levels throughout pregnancy. Initiating and maintaining a low-Phe diet before conception and throughout pregnancy helps keep blood Phe levels within the recommended range, supporting healthy pregnancy outcomes. Women with unplanned pregnancies face a heightened risk of MPKU syndrome^1^^,^^2^ Despite years of efforts, MPKU syndrome remains an important health issue in women with PKU, with the frequency of unplanned pregnancies still high^29^^,^^30^

Reliable global data on the prevalence of MPKU syndrome are lacking. One study from the United States estimated that the number of babies at risk of MPKU-related disabilities is almost as high as the number of babies born with PKU^31^ Two cases of MPKU syndrome in transitioned individuals were caused by an undiagnosed hyperphenylalaninemia in their mothers, diagnosed after giving birth to a child with MPKU syndrome. High prevalence (25%) of children with MPKU syndrome born to the transitioned mothers in the years preceding the transition may indicate that pre-transition care had been inadequate to prevent MPKU syndrome and underscores the critical need for structured reproductive health counseling in adult PKU care.

During the follow-up period of this study, there were two pregnancies in the transitioned cohort: 1 planned in an individual with mild hyperphenylalaninemia who, despite multiple attempts to contact, was LTFU, and the second, which was unplanned, in an individual who had been regarded as LTFU in the pediatric center, with poor metabolic control and a history of non-adherence who contacted our center after the realization that she was pregnant. To put this number in context, up to half of pregnancies in PKU women may be unplanned despite intensive education efforts^29^ In the cited report from Germany, 3 out of 25 pregnancies were unplanned and resulted in a mother giving birth to a child with MPKU syndrome^4^ It is crucial that our efforts are aimed at ensuring the number of unplanned pregnancies is as low as possible to reduce the risk of MPKU syndrome. The number of unplanned pregnancies and any births to a baby with MPKU syndrome should be regarded as critical safety outcomes in our future reports, establishing critical benchmarks for the adult program's success. Finally, women with PKU in the childbearing age who have been LTFU in any pediatric center should be reached proactively to avoid an undesirable scenario reported above.

This study has several important limitations. Firstly, its design lacks a control group. However, the implemented strategy, with comparisons between the two time periods ‒ pre- and post-transition ‒ is an established method for reporting transition results in PKU^4^^,^^5^^,^^13^ Although suitable for this initial descriptive report, the retrospective analysis of clinical data may have introduced information bias because of incomplete documentation in medical records. Additionally, a 1-year time frame for the transition program is relatively short to draw wider conclusions about its success or failure; thus, a longer follow-up is needed. Nevertheless, we believe it is enough to report initial data from the region's first cohort. The single-center design remains a limitation, although this is partly mitigated by the fact that adult PKU centers are only now being established in Poland, making broader comparisons currently unfeasible. A selection bias caused by prioritization in the transition of older patients with LTPKU, and possibly of patients and caregivers with higher motivation (due to the need to organize the first visit themselves), should be considered when analyzing our results. Finally, multiple univariate comparisons performed during the analysis of data were exploratory and hypothesis-generating. Thus, the statistical significance of findings should be interpreted with caution. A key strength of this study is its large cohort, comprising all adults with PKU treated at our center.

## Conclusions

In this report, we summarized one year of pioneering efforts in the transition of adults with PKU in Poland. Key innovations in the Polish context of transition programs included a structured transition protocol from pediatric to adult metabolic care, involvement of multidisciplinary stakeholders, including a nutritionist and a psychologist, an education initiative on PKU and MPKU syndrome, and comprehensive laboratory and radiological screening for comorbidities.

The Phe-control in the studied cohort remained inadequate and did not improve after the transition, highlighting that maintaining long-term adherence to the restrictive diet in adults with PKU is challenging. As the observation period in this study was relatively short, a longer follow-up is needed to draw wider conclusions about the impact of transition on the metabolic control of Phe.

The frequency of unplanned pregnancies and MPKU syndrome among children of transitioned individuals was alarmingly high and may indicate that pre-transition care had been inadequate to prevent it, representing a major adult PKU healthcare crisis. The need for structured reproductive health counseling in adult PKU care is critical and must be prioritized within adult metabolic care. These preventable pre-transition outcomes should serve as a benchmark against which the adult program's success in preventing MPKU should be measured.

The adult population with PKU remains under-researched, and the effects of PKU on the aging process are not well understood. Further investigation is needed to evaluate the burden of comorbidities and loss of quality of life, support lifelong treatment and its sustainability, and provide a safe environment for pregnancy.

## Declaration of generative AI and AI-assisted technologies in the manuscript preparation process

During the preparation of this work, the authors did not use any generative AI and AI-assisted technologies

## Data availability statement

The datasets generated and/or analyzed during the current study are available from the corresponding author on reasonable request.

## Authors’ contributions

Michal Kania: Conceptualization; data curation; formal analysis; investigation; methodology; project administration; validation; visualization; writing-original draft; writing-review and editing.

Martyna Pietruś: Data curation; investigation; methodology; writing-original draft, writing-review and editing.

Łukasz Pawliński: Data curation; investigation; methodology; writing-original draft; writing-review and editing; project administration; resources; methodology; writing-original draft; writing-review and editing.

Karolina Orchel-Szastak: Data curation; resources; writing-original draft; writing-review and editing.

Przemko Kwinta: Methodology; project administration; resources; writing-original draft; writing-review and editing.

Maciej T. Małecki: Methodology; project administration; resources; writing-original draft; writing-review and editing.

## Funding and disclosures

This study did not require additional funding.

## Conflicts of interest

The authors declare that the research was conducted in the absence of any commercial or financial relationships that could be construed as a potential conflict of interest.
